# Hypokalemia-Induced Arrhythmia: A Case Series and Literature Review

**DOI:** 10.7759/cureus.22940

**Published:** 2022-03-07

**Authors:** May Thu Kyaw, Zay Maung Maung

**Affiliations:** 1 Internal Medicine, University of Medicine 2, Yangon, MMR; 2 Cardiology, Heart and Vascular Center, Victoria Hospital, Yangon, MMR; 3 Internal Medicine, University of Medicine, Magway, MMR

**Keywords:** arrhythmia, ecg, cell membrane excitability, hypokalemia, potassium

## Abstract

Electrolyte imbalances, particularly potassium disorders, are common in clinical practice. Potassium homeostasis plays a key role in regulating cell membrane excitability. Hypokalemia usually presents with cardiovascular and neuromuscular abnormalities. Hypokalemia can lead to clinically significant life-threatening arrhythmia. Typical electrocardiographic (ECG) features of hypokalemia include widespread ST depression, T wave inversion, and prominent U waves. However, hypokalemia may present with different types of arrhythmia, such as premature ventricular contractions, ventricular fibrillation, atrial fibrillation, and torsade de pointes. Thus, clinicians should be familiar with ECG manifestations of potassium disorders that may warrant timely diagnosis and effective management. Herein, we report three patients with arrhythmia who were found to have typical ECG characteristics of hypokalemia after resolution of arrhythmia and later proved to have low serum potassium levels.

## Introduction

The normal serum potassium concentration ranges from 3.5 to 5.3 mmol/L. Hypokalemia is considered to be severe if the serum potassium level is lower than 2.5 mmol/L. It is estimated that the total body potassium storage in a 70 kg person is 3500 mmol, 98% of which is intracellular and only 2% is extracellular [1]. Thus, a mild decrease in serum potassium level can be associated with significantly depleted total body potassium pool. A reduction in serum potassium level of approximately 0.3 mmol/L corresponds to a 100 mmol deficit in the total body potassium pool [2]. Potassium (K+) is the major cation in the intracellular fluid and plays a central role in maintaining cell membrane excitability. A low serum potassium level increases the transmembrane electrochemical gradient, thus hyperpolarizing the cells and impairing depolarization and contraction. This can lead to a severe disturbance in cardiac conduction pathways. Although hypokalemia usually presents with muscle weakness, arrhythmia may sometimes be the first clinical presentation of severe hypokalemia.

## Case presentation

Case 1: atrial fibrillation due to hypokalemia

**Figure 1:**
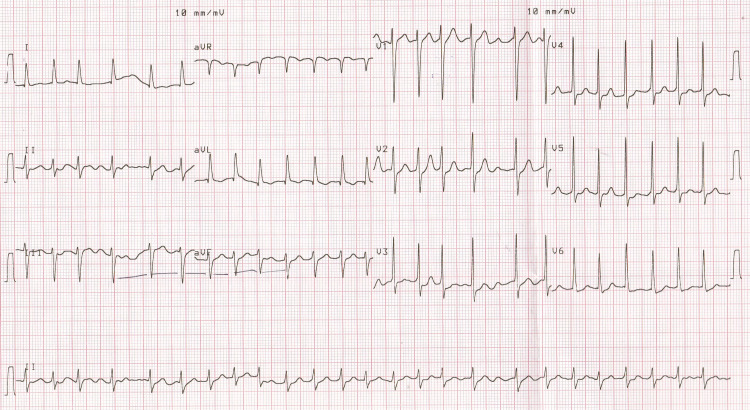
Atrial fibrillation with rapid ventricular response.

**Figure 2:**
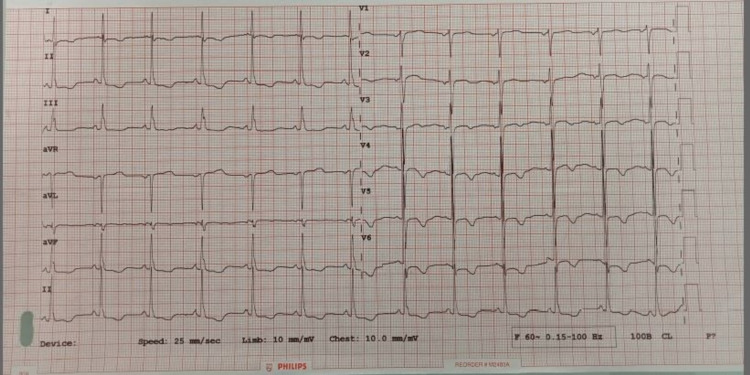
ECG showing prolonged QT interval, ST depression, and T inversion.

An 80-year-old woman presented to the emergency department with a complaint of chest discomfort and palpitation. She had a history of hypertension, type 2 diabetes mellitus, and chronic obstructive airway disease. On initial assessment, she was dyspneic with a blood pressure of 82/46 mmHg and oxygen saturation of 93% on room air. Neurologic examination was normal without significant muscle weakness. Electrocardiogram (ECG) showed atrial fibrillation with rapid ventricular response (Figure 1). After intravenous amiodarone infusion, the patient showed marked clinical improvement. ECG after stabilization showed sinus rhythm with features of hypokalemia (Figure 2).

**Figure 3:**
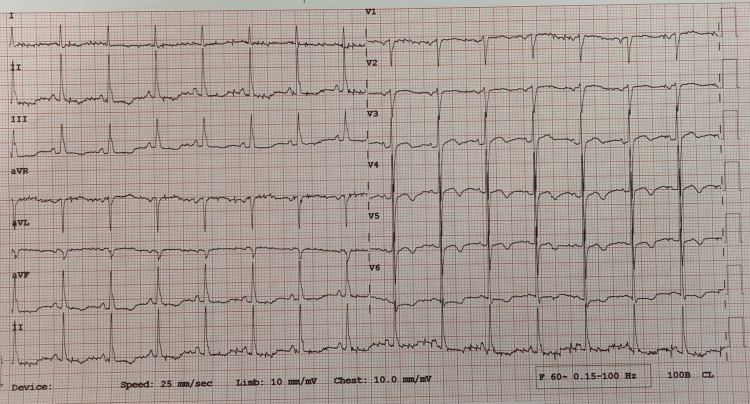
ECG showing ST depression and T inversion.

Serum electrolyte results showed significant hypokalemia (K = 2.4 mmol/L, normal = 3.5-5.3 mmol/L) with borderline hypomagnesemia (Mg = 1.49 mg/dL, normal = 1.50-2.60 mg/dL). She was treated with potassium supplement (both intravenous and orally). When the serum potassium level reached 4.56 mmol/L, the ECG was rechecked and showed the continued presence of ST depression and T inversion, indicating possible underlying associated myocardial ischemia (Figure 3).

Case 2: supraventricular tachycardia due to hypokalemia

A 38-year-old woman presented to the clinic with palpitation and a feeling of “tiredness” in the chest for one day. She had no history of chest pain or muscle weakness. However, she noted a recent onset of polyuria (seven to eight times per day), although she could not remember the estimated amount. She denied any history of previous heart disease, hypertension, diabetes, or kidney disease. However, she had a history of childhood asthma which had been stable for years without treatment. She also had no history of taking regular medications, including diuretics or herbal medications. There was no family history of sudden cardiac death. On assessment, she was dyspneic with a blood pressure of 150/90 mmHg, heart rate of 164 beats per minute (bpm), oxygen saturation of 100% on room air, and temperature of 36.5°C. The capillary blood glucose level was 112 mg/dL. Physical examination revealed normal first and second heart sounds with no added sound. Lungs were clear and both abdominal and neurologic examinations were normal. There were no clinical features suggestive of hypothyroid, hyperthyroid, or Cushing’s syndrome.

**Figure 4:**
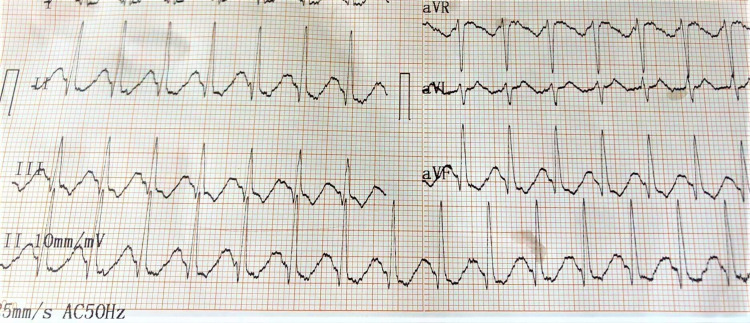
ECG showing supraventricular tachycardia.

**Figure 5:**
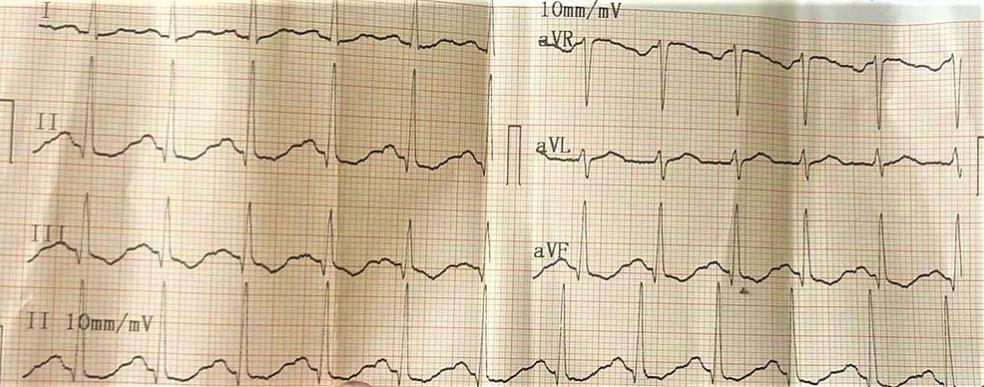
ECG showing ST depression, small T wave, prominent U wave, and prolonged QT interval.

The initial ECG showed supraventricular tachycardia with a ventricular rate of 164 bpm (Figure 4). Vagal maneuvers were tried but no response was elicited. Therefore, the patient was administered intravenous amiodarone 150 mg + normal saline 100 mL over 30 minutes. ECG rechecked after amiodarone infusion showed features of hypokalemia such as ST depression, small T wave, prominent U waves, and prolonged QT interval (Figure 5).

**Figure 6:**
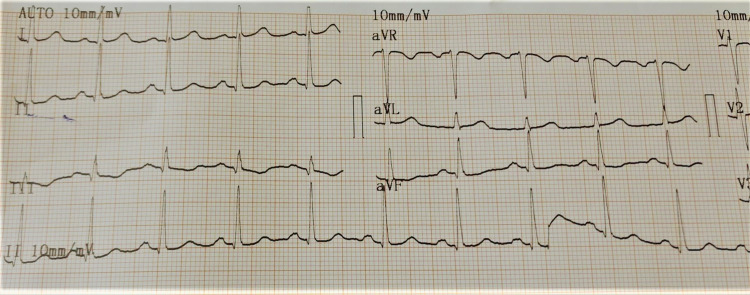
ECG showing resolution of hypokalemia features.

Accordingly, serum potassium was urgently checked, which showed severe hypokalemia (K = 2.7 mmol/L). Supplemental potassium chloride was administered (both intravenous and orally). The patient’s serum potassium level increased to 3.87 mmol/L and a repeat ECG showed resolution of hypokalemia features (Figure 6).

Case 3: ventricular bigeminy due to hypokalemia

**Figure 7:**
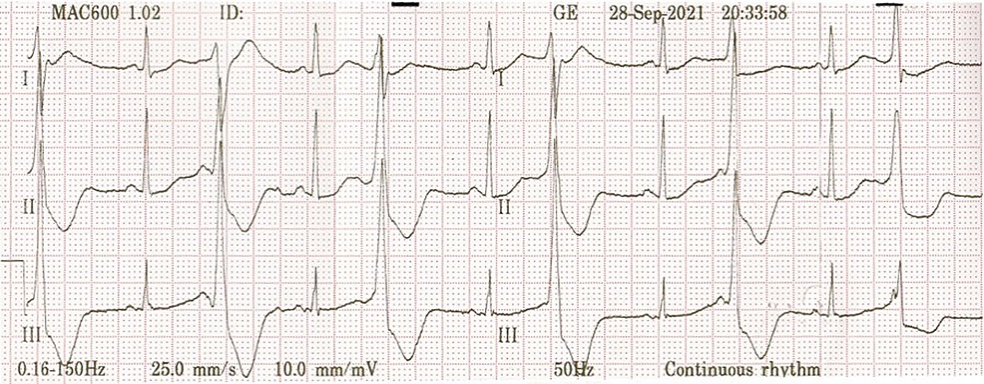
ECG showing ventricular bigeminy.

A 36-year-old woman came to the emergency department with a complaint of syncopal attack. She was a chronic alcoholic and binge drinker. She also noticed occasional palpitation and proximal muscle weakness. She denied chest pain or dyspnea. She had no history of hypertension or diabetes. She denied having a history of diarrhea, vomiting, laxative or diuretic misuse, or licorice ingestion. On assessment, she was stable with a blood pressure of 110/70 mmHg, heart rate of 92 bpm, and oxygen saturation of 98% on room air. ECG showed ventricular bigeminy (Figure 7).

**Figure 8:**
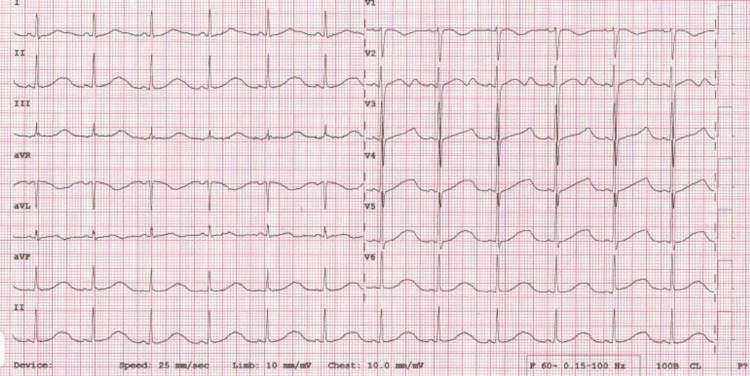
ECG showing prolonged QT interval.

The patient’s serum potassium level was significantly low (2.59 mmol/L) and borderline hypomagnesemia was also noted (Mg = 1.57 mg/dL). After administering the first dose of intravenous potassium supplement (KCl 40 mmol), the ECG was rechecked, showing the disappearance of ventricular bigeminy with the appearance of a typical hypokalemic ECG pattern such as prolonged QT interval (Figure 8).

Further doses of intravenous potassium supplements were planned for administration to the patient. However, due to personal reasons, she wanted to be discharged against medical advice. She was given oral potassium and magnesium supplements and planned to follow-up to recheck the electrolyte results. Unfortunately, the patient also missed follow-up.

## Discussion

Hypokalemia usually presents with muscle weakness and abnormalities of cardiac rhythm. The main etiologies of hypokalemia can be categorized into three groups: increased loss (renal or gastrointestinal), intracellular shift (due to medications or hormonal abnormalities), and reduced dietary K+ intake [3]. Characteristic ECG changes of hypokalemia include dynamic changes in T wave morphology (flattening and inversion of T waves) in mild hypokalemia, followed by prolonged QT interval, prominent U wave, and mild ST segment depression in severe cases. Moreover, hypokalemia can present with various patterns of arrhythmia such as premature ventricular complexes, atrial fibrillation, atrial flutter, supraventricular tachycardia, and, in the worst cases, torsade de pointes, ventricular tachycardia, and ventricular fibrillation, which can be life-threatening [4]. Moderate hypokalemia (2.5-3 mmol/L) is associated with cardiac arrhythmia and other muscular disorders. Consequently, severe hypokalemia (<2.5 mmol/L) is life-threatening and may lead to cardiac arrest [5].

Cardiac muscles contract via the process called excitation-contraction coupling. The action potential (AP) starts from the sinoatrial node and propagates the whole heart through the atrioventricular node, His bundle, and Purkinje fibers. The AP is determined by the transcellular electrochemical gradient, which is mainly regulated by opening and closing of various ion channels. Any disturbances in AP generation and/or conduction can lead to arrhythmia [6]. Intracellular potassium concentration (K+) is 30-40 times higher than the extracellular concentration. The serum K+ concentration is strictly regulated within the range of 3.5-5.3 mmol/L by the electrophysiological activity of cell membrane sodium/potassium adenosine triphosphatase (ATPase) (Na+/K+ ATPase) activity. In cardiac myocytes, Na+/K+ ATPase is the major Na+ efflux mechanism. Na+/K+ ATPase is an energy- and voltage-dependent cell membrane ion transporter that pumps three intracellular Na+ ions out of the cell in exchange for two extracellular K+ ions into the cell, causing a net outward current. The activity of Na+/K+ ATPase is maintained by extracellular K+ concentration, intracellular Na+ concentration, and membrane AP [7].

Hypokalemia induces arrhythmia by two distinct mechanisms: direct inhibition of K+ conductance and indirect suppression of Na+/K+ ATPase activity. The latter results in intracellular Na+ accumulation. High intracellular Na+ reduces outward calcium (Ca+) current via Na/Ca exchanger, and as a result, increased intracellular Ca+ content and delayed after-depolarization occur. Cytosolic Ca+ activates Ca/calmodulin-dependent protein kinase II (CaMKII) by phosphorylation of the ryanodine receptor. Activated CaMKII stimulates late Na+ current (INa) and I-type Ca+ current (ICa-L) as well. This positive feedforward loop further increases the intracellular concentration of Ca+. High Ca+ is associated with pacemaker abnormalities. Subsequently, the final result is an altered repolarization process and prolonged AP duration, triggering early and delayed after-depolarization and risk of various types of arrhythmia [8,9].

In 2019, Phillips et al. conducted a longitudinal, multicenter retrospective cohort study that showed that hypokalemia <3.5 mmol/L was associated with an increased risk of medically treated arrhythmia in intensive care unit patients [10]. The Rotterdam Study concluded that independent of age, sex, serum magnesium concentration (Mg+), and other potential confounding factors, hypokalemia (<3.5 mmol/L) was associated with a higher risk for atrial fibrillation (hazard ratio: 1.63, 95% confidence interval 1.03-2.56) when compared with normokalemia [11].

Chronic hypokalemia is better tolerated. Generally, parenteral K+ supplement is indicated if there is severe (<2.5 mmol/L) or moderate hypokalemia with electrical instability (cardiac arrhythmia). The rate of intravenous K+ administration should not exceed 10-20 mmol/h in adults and 0.4 mmol/kg/h in children [12]. The 2000 American Heart Association guidelines for cardiopulmonary resuscitation and emergency cardiovascular care recommend that in patients with hypokalemia-induced malignant ventricular arrhythmia or cardiac arrest, an empirical intravenous injection of 2 mmol of potassium should be administered in the first one minute, followed by an intravenous infusion of 10 mmol of potassium over five to 10 minutes [13]. The maximum potassium supplement dose is as high as 480 mmol/24 h, and in patients with underlying heart block or renal insufficiency, it is up to 120 mmol/24 h [2]. In 2019, Du et al. conducted an experiment in animal models that showed that the strategy of tailored rapid potassium supplementation is a safe and efficient option for reversing life-threatening arrhythmia due to severe hypokalemia. However, more studies are required to draw a definitive conclusion [12].

However, a normal serum potassium level after potassium replacement does not necessarily indicate potassium store repletion. The patient might still be experiencing depletion of intracellular potassium. Therefore, to ensure that a stable potassium level is achieved, monitoring signs and symptoms of recurrent hypokalemia, ECG, and the serum potassium level for 24 h are recommended [2].

## Conclusions

Hypokalemia should be suspected in any patient presenting with arrhythmia. The early diagnosis of hypokalemia based on clinical presentation and changes in ECG is extremely important for timely intervention. Therefore, for the effective management of arrhythmia in acute care settings, clinicians should be familiar with different ECG manifestations of hypokalemia. Moreover, after potassium replacement, even with normal serum potassium levels, clinicians should be aware of intracellular potassium depletion, which may warrant monitoring for recurrent hypokalemia and further potassium supplementation.

## References

[1] Palmer BF, Clegg DJ (2016). Physiology and pathophysiology of potassium homeostasis. Adv Physiol Educ.

[2] Abdulfattah O, Rahman EU, Alnafoosi Z, Schmidt F (2018). Severe hypokalemia with cardiac arrest as an unusual manifestation of alcoholism. J Community Hosp Intern Med Perspect.

[3] Tse G, Li KH, Cheung CK (2021). Arrhythmogenic mechanisms in hypokalaemia: insights from pre-clinical models. Front Cardiovasc Med.

[4] Chua CE, Choi E, Khoo EY (2018). ECG changes of severe hypokalemia. QJM.

[5] Chen H, Chatelain FC, Lesage F (2014). Altered and dynamic ion selectivity of K+ channels in cell development and excitability. Trends Pharmacol Sci.

[6] Varró A, Tomek J, Nagy N, Virág L, Passini E, Rodriguez B, Baczkó I (2021). Cardiac transmembrane ion channels and action potentials: cellular physiology and arrhythmogenic behavior. Physiol Rev.

[7] Weiss JN, Qu Z, Shivkumar K (2017). Electrophysiology of hypokalemia and hyperkalemia. Circ Arrhythm Electrophysiol.

[8] Oshita K, Kozasa Y, Nakagawa Y (2019). Overexpression of the HCN2 channel increases the arrhythmogenicity induced by hypokalemia. J Physiol Sci.

[9] Faggioni M, Knollmann BC (2015). Arrhythmia protection in hypokalemia: a novel role of Ca2+-activated K+ currents in the ventricle. Circulation.

[10] Phillips CT, Wang J, Celi LA, Zhang Z, Feng M (2019). Association of hypokalemia with an increased risk for medically treated arrhythmias. PLoS One.

[11] Krijthe BP, Heeringa J, Kors JA, Hofman A, Franco OH, Witteman JC, Stricker BH (2013). Serum potassium levels and the risk of atrial fibrillation: the Rotterdam Study. Int J Cardiol.

[12] Du Y, Mou Y, Liu J (2019). Efficiency evaluation and safety monitoring of tailored rapid potassium supplementation strategy for fatal severe hypokalemia. Exp Ther Med.

[13] (2000). Guidelines 2000 for cardiopulmonary resuscitation and emergency cardiovascular care. Part 8: advanced challenges in resuscitation: section 1: life-threatening electrolyte abnormalities. The American Heart Association in collaboration with the International Liaison Committee on Resuscitation. Circulation.

